# The Mental Health Impacts of COVID-19 on Pediatric Patients Following Recovery

**DOI:** 10.3389/fpsyg.2021.628707

**Published:** 2021-06-29

**Authors:** Dong Liu, Wenjun Liu, Marcus Rodriguez, Jie Zhang, Fuhai Zhang

**Affiliations:** ^1^Department of Communication, Renmin University of China, Beijing, China; ^2^Pitzer College, Claremont, CA, United States; ^3^Boston Child Study Center, Los Angeles, CA, United States; ^4^Wuhan Children's Hospital, Tongji Medical College, Huazhong University of Science and Technology, Wuhan, China; ^5^School of Education, Hebei Normal University, Shijiazhuang, China

**Keywords:** COVID-19, PTSD, anxiety, depression, children

## Introduction

According to data from American Academy of Pediatrics, over 3.1 million children have tested positive for COVID-19 by March 01 in the U.S. Although children experience lower rates of severe symptoms and have higher rates of asymptomatic infection as compared to adults, the mental health of children is more vulnerable than that of older individuals (Loades et al., 2020). Although some studies reported mental health outcomes of the general children during the pandemic (Duan et al., 2020), evidence regarding mental health of children infected by COVID-19 are largely understudied. Children or adolescents infected with COVID-19 may experience a series of physical symptoms—often including fever and respiratory distress. Previous studies showed that children might experience trauma, poor sleep, and withdrawn behaviors during the pandemic (Douglas et al., 2009). In the COVID-19 pandemic, cluster infection is more likely to occur within the families of infected children (Dong et al., 2020), which increases the risk of parental infections— possibly leading to a lack of mental and emotional support from the child's parents (Holmes et al., 2020). It is possible that children infected by COVID-19 may experience similar psychological problems. Due to the large number of infected children worldwide, it is urgent to know the emotional and mental health outcomes of infected children.

To the best of our knowledge, no studies have yet reported the prevalence of symptoms associated with anxiety, depression, and PTSD among recovered, pediatric COVID-19 patients. There is also a dearth of information regarding the risk factors associated with mental distress among pediatric patients. It is urgent for researchers to clarify these issues to better understand the mechanisms by which mental health problems develop in pediatric patients (Prime et al., 2020). In this study, we examine the mental health outcomes of 38 pediatric patients who were admitted to the Wuhan Children's Hospital with cases of COVID-19.

## Methods

### Study Design

The following study—utilizing a cross-sectional survey design—was conducted between June 30 and July 15, 2020 and was approved by the research ethics committee in Renmin University of China on May 20, 2020. Following approval, nurses at the Wuhan Children's Hospital were presented with the survey materials, which they described to the parents of candidate patients before obtaining their oral consent. Two separate online survey links were sent to the parent and child participants. The children only needed to report their gender, age, and psychological symptoms; all other information was obtained from their parents.

### Participants

Nurses reviewed the electronic medical records of all COVID-19 patients under the age of 18. Inclusion criteria for participants included: (1) inpatient hospitalization; (2) symptoms consistent with COVID-19; and (3) an age between 5 and 18 years. Eligible participants had all experienced a full recovery and were subsequently discharged from the hospital— with a median of 110 days since discharge (ICQ [102, 129]) and an average hospital stay of 11 days (ICQ [7, 15.5]). The date of hospital admission ranged from January 27, 2020 to March 28, 2020, and the date of discharge ranged from February 2, 2020 to April 30, 2020. Nurses distributed online questionnaires to 112 patients, yielding 77 responses; however, only 38 pairs of parents and children completed the entire questionnaire.

### Measures

#### The Screen for Child Anxiety Related Emotional Disorders (SCARED)

SCARED (Birmaher et al., 1997, 1999) is a self-report questionnaire which assesses anxiety disorder symptoms in children and adolescents under the age of 18. The scale is composed of 41 items, and children are asked to report the frequency of each symptom on a 3-point-scale: 0 (almost never), 1 (sometimes), or 2 (often). The reliability was 0.993.

#### The Children's Depression Inventory (CDI)

CDI is a widely used self-report inventory for assessing depression in school-aged children (Saylor et al., 1984). It contains 27 items which each consist of three choices that are graded in order of increasing severity, from 0 to 2. Children select the sentence from each group which best describes themselves during the prior two weeks period. The reliability was 0.954.

#### The UCLA Child/Adolescent PTSD Reaction Index for DSM-5

This is the revised UCLA Child/Adolescent PTSD Reaction Index from DSM-IV (Kaplow et al., 2020). The new version is a semi-structured interview which assesses a child's traumatic history and the full range of DSM-5 diagnostic criteria for PTSD among school-age children and adolescents. The reliability was 0.989.

#### Illness Severity and Symptoms

The questionnaire elicited parental reports of key clinical variables, including: the severity of COVID-19 pneumonia (asymptomatic, mild, moderate, severe, or critically ill); the date of symptom onset; date of hospital admission; date of discharge; and ongoing symptoms following hospital discharge (e.g., cough, respiratory distress, chest pain, dizziness, fatigue, dyspnea, etc.).

#### Parental Ignorance

Parents were asked the extent to which they are unaware of their child's daily activities. We constructed a brief, 5-item parental ignorance scale for the current study (e.g., “I check the symptoms of my children daily”). The reliability was 0.787.

#### Peer Rejection

Parents reported peer rejection according to the following single-item prompt: “My child was rejected by his/her friends.”

### Other Measures

In addition to the aforementioned metrics, parents were also asked to report: the mental health status of their children prior to infection; whether or not they themselves contracted COVID-19; the number of family members who contracted COVID-19; whether any of their family members died from COVID-19; and the amount of time the child was separated from his or her parents and other family members.

### Analysis Strategy

We performed analyses on three separate mental health outcomes: anxiety, depression, and PTSD. Descriptive statistics of demographic variables, clinical features, anxiety, depression, and PTSD are presented below. We also conducted the logistic regression to explore the risk factors for pediatric patients' mental problems. Limited by the sample size, we also reported the marginally significant results.

## Results

### Demographic Profile of the Sample

**Figure 1:**
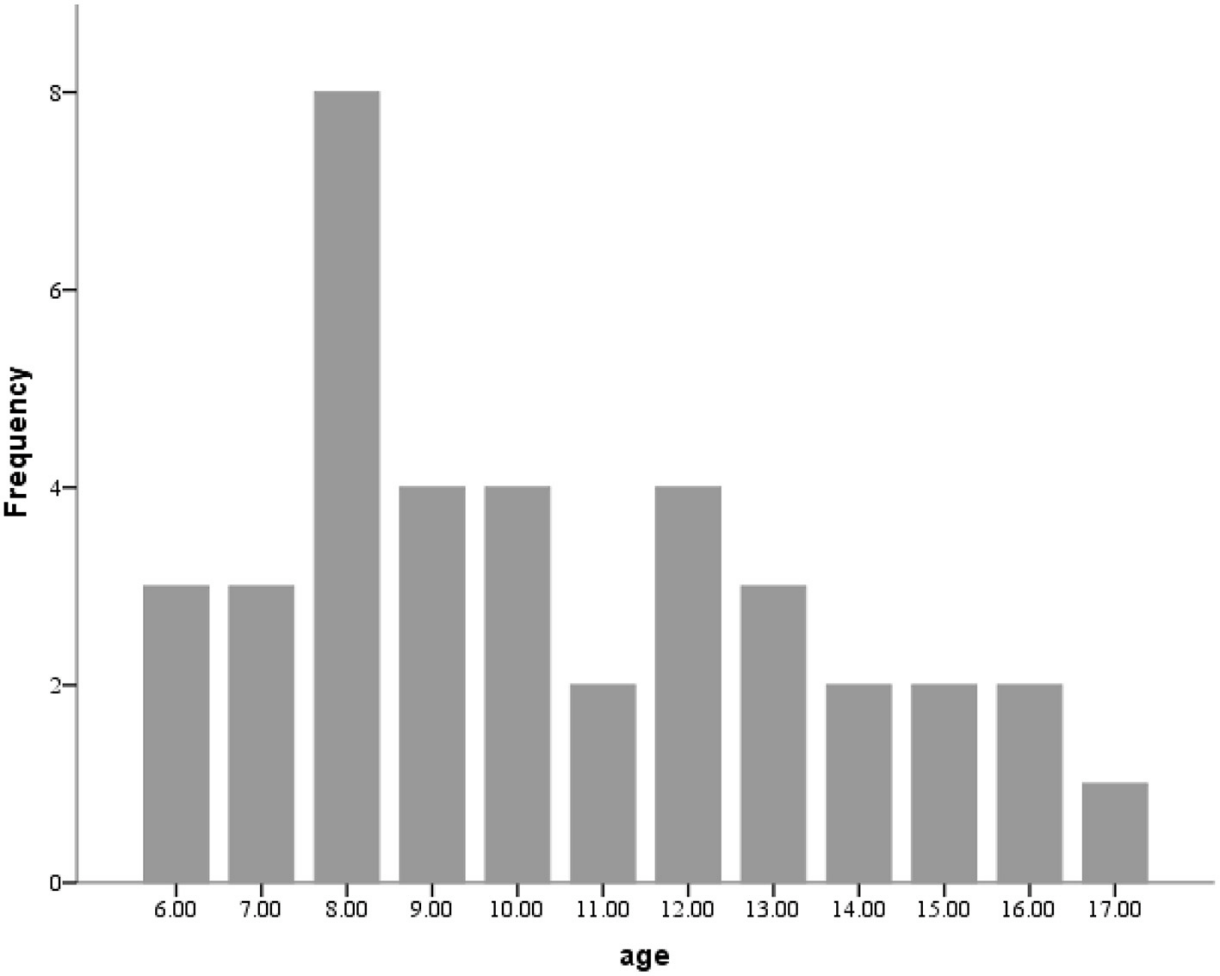
Age distribution of sample.

Thirty-eight pairs of parents and children completed the survey. The median age of the child cohort was 10 years (ICQ [8, 13]) and 14 (36.8%) of the participants were female. 24 (63.2%) children had at least one other family member infected with COVID-19, and 10 (26.3%) had family members die as a result of COVID-19 infection. A comprehensive compendium of demographic information is reported in Table 1. The age distribution can be found in Figure 1.

**Table 1:** Characteristics of pediatric patients with COVID-19.

**Variables**	***N* (%)**
**Gender**	
Male	23 (60.5%)
Female	15 (39.5%)
**Marital status**	
Married	31 (81.6%)
Divorced	7 (18.4%)
**COVID-19 severity**	
Asymptomatic	3 (7.9%)
Mild	17 (44.7%)
Moderate	16 (42.1%)
Severe	2 (5.3%)
**COVID-19 symptoms**	
Dizziness	4 (10.5%)
Headache	5(13.2%)
Loss of appetite	6 (15.8%)
Chest distress	4 (10.5%)
Olfactory dysfunctions	3 (7.9%)
Fatigue	8 (21.1%)
Cough	7 (18.4%)
Diarrhea	10 (26.3%)
Vomit	1 (2.6%)
**Family infection (except children)**	
0	14 (36.8%)
1	11 (28.9%)
2	11 (28.9%)
3	1 (2.6%)
4	1 (2.6%)
**Family member death**	
Yes	10 (26.3%)
No	28 (73.7%)
**Mental health before infection**	
Very Good	11 (28.9%)
Good	8 (21.1%)
Neutral	8 (21.1%)
Bad	10 (26.3%)
Very Bad	1 (2.6%)

### COVID-19 Symptoms After Hospital Discharge

A large proportion of the children suffered only mild (17, 44.7%) or moderate (16, 42.1%) symptoms. Only 3 (7.9%) patients were completely asymptotic, and 2 patients (5.3%) reported severe symptoms. Unlike adult patients, diarrhea (10, 26.3%), fatigue (8, 21.1%), and cough (7, 18.4%) were the most common symptoms for children and adolescents following hospital discharge. Some symptoms which are prevalent in adult COVID-19 patients were rare in children, including: respiratory distress (0, 0%), nausea (0, 0%), and vomiting (1, 2.6%). Additionally, some patients reported a loss of appetite (6, 15.8%) and olfactory dysfunction (3, 7.9%).

### Descriptive Analysis of Mental Health Outcomes

Seven (18.4%) participants were provisionally diagnosed with clinically significant symptoms of PTSD, 12 (31.6%) were categorized as having significant symptoms of anxiety, and six (15.8%) were categorized as having significant symptoms of depression. The median scores for CDI, SCARED, and UCLA PTSD-RI were 9 (ICQ [6, 15]), 8.5 (ICQ [1, 37.5]), and 10.5 (ICQ [0, 27]), respectively. The median score for the social withdrawal scale was 3.37 (ICQ [2.75, 3.5]).

### Risk Factors of Mental Health Outcomes

Among all COVID-19 symptoms, only vomiting was associated with anxiety (*r* = 0.416, *p* = 0.016), depression (*r* = 0.406, *p* = 0.011) and PTSD (*r* = 0.454, *p* = 0.004). All other correlation coefficients between COVID-19 symptoms and psychiatric symptoms were not significant. Peer rejection was marginally associated with depression (*r* = 0.264, *p* = 0.109), anxiety (*r* = 0.274, *p* = 0.096), and PTSD (*r* = 0.264, *p* = 0.110), while social withdrawal was associated with depression (*r* = 0.285, *p* = 0.083), anxiety (*r* = 0.314, *p* = 0.055), and PTSD (*r* = 0.313, *p* = 0.056). Notably, poor mental health status prior to COVID-19 infection was associated with an increase in mental health problems following infection (anxiety, *r* = −0.266, *p* = 0.106; depression, *r* = −0.298, *p* = 0.069; PTSD, *r* = −0.350, *p* = 0.031; see Table 2). Peer rejection was associated with anxiety (*r* = 0.274, *p* = 0.096), depression (*r* = 0.264, *p* = 0.109), and the PTSD score (*r* = 0.264, *p* = 0.110). Parental infection was positively associated with parental ignorance (*r* = 0.287, *p* = 0.081), which was negatively associated with children's social withdrawal (*r* = −0.311, *p* = 0.057).

**Table 2:** Observed zero-order correlations between variables.

	**1**	**2**	**3**	**4**	**5**	**6**	**7**	**8**	**9**	**10**	**11**	**12**	**13**
(1). Gender	1												
(2). Age	0.231	1											
(3). Covid-19 Severity	0.022	0.233	1										
(4). Mother separation	−0.023	−0.116	0.195	1									
(5). Parental infection	−0.184	0.128	0.012	−0.26	1								
(6). Family death	−0.129	−0.04	0.123	0.145	−0.147	1							
(7). Family infection	0.227	0.131	−0.107	0.189	0.062	0.211	1						
(8). Peer rejection	0.235	0.18	0.147	−0.055	0.443^**^	0.133	−0.146	1					
(9). Mental status (before)	−0.355^*^	−0.057	−0.061	0.256	−0.155	−0.182	0.28^+^	−0.647^**^	1				
(10). Parental ignorance	−0.09	−0.177	−0.284^+^	−0.084	0.287^+^	0.125	0.094	−0.153	0.006	1			
(11). Withdrawal	0.199	0.187	0.164	−0.008	0.035	0.118	−0.292^+^	0.456^**^	−0.277^+^	−0.311^+^	1		
(12). Anxiety	0.182	−0.138	0.018	−0.145	0.093	0.061	−0.186	0.264^+^	−0.266^+^	−0.075	0.314^+^	1	
(13). Depression	0.085	−0.061	0.141	−0.108	0.153	0.055	−0.3^+^	0.274^+^	−0.298^+^	−0.064	0.285^+^	0.818^**^	1
(14). PTSD	0.113	−0.132	0.087	−0.142	0.053	0.013	−0.275^+^	0.264^+^	−0.350^*^	−0.073	0.313^+^	0.955^**^	0.869^**^
Mean	1.38	10.39	2.53	8.31	0.42	0.27	1.02	2.31	3.31	25.95	23.39	18.69	11.80
SD	0.49	3.09	0.73	15.59	0.50	0.44	0.94	0.95	1.26	3.17	3.41	24.13	12.39

*1 refers to male, 2 refers to female*.

+ *p < 0.10;*

* *p < 0.05;*

** *p < 0.01*.

We conducted logistic regression with depression, anxiety, and PTSD as dependent variables. Peer rejection was marginally associated with PTSD (OR, 29.29, 95% CI, [0.94, 908.67]), while social withdrawal was associated with anxiety (OR, 4.61, 95% CI, [0.95, 22.44]).

## Discussion

The results of this study suggest that a significant proportion of pediatric patients suffer from mental disorders following COVID-19 recovery. Although the study cohort's rate of depression was not higher than that of normal primary students in Wuhan (15.8 vs. 22.6%), their anxiety rate was nearly double that of normal students (31.6 vs. 18.9%) (Xie et al., 2020). Overall, these rates are higher than the rates of psychiatric symptom observed in the adult patient population (31.6 vs. 10.4% for anxiety, and 18.4 vs. 12.4% for PTSD) (Liu et al., 2020). Because children with COVID-19 face risks of long-lasting physical symptoms and discrimination (more risks), their high anxiety level may keep for a longer time than normal children.

Although most children experience milder physical symptoms of COVID-19 than adults, pediatric patients may face more psychological threats following hospital discharge. Physical symptoms of COVID-19 may not be the main threats for children. Most of the pediatric patients in our sample reported at least one physical symptom of COVID-19— with diarrhea, sore throat, cough, and headache being the most reported symptoms. However, only vomiting was associated with mental health outcomes (anxiety, depression, and PTSD).

Peer rejection was the main risk factor for mental health outcomes of COVID-19 infected children. Socially withdrawn children are expected to experience a series of negative developmental outcomes, including emotional problems (e.g., anxiety and depression) and social adjustment difficulties (Liu et al., 2015). Notably, family cluster infection was prevalent in COVID-19 patients. On the one hand, parental infection leads to stigma and peer rejection; on the other hand, parental ignorance and maternal warmth loss may leave children without protection. Out data suggested that a significant proportion of infected children may withdraw from their peers socially.

### Limitations

Limitations of this study include the cross-sectional survey design and a small sample size, which limits the overall power of statistical analyses. Meta-analyses of individual pediatric data should be conducted in the future to enhance the power. We also recommend future studies to collect longitudinal and developmental data for recovering children of COVID-19 because the effects may last for years for children after the pandemic (Wade et al., 2020). Secondly, only one-third of recovered and discharged COVID-19 patients from the Wuhan Children's Hospital agreed to participate into the study— resulting in a possible self-selection bias. Finally, all respondents were Chinese children, which may limit the generalizability of the findings.

## Conclusions

Pediatric patients are psychologically more vulnerable than adults. Compared with adults, children face more threats (duration of quarantine and lack of necessary supplies, lack of information) during the pandemic (Loades et al., 2020). Parental infection can have a profound impact on parent-child and peer relationships, and it was found that strong, high-quality relationships between youths and their parents and peers were important resilience factors in protecting the mental health of recovered pediatric COVID-19 patients. Hence, parents and caregivers are encouraged to cultivate supportive environments for their children, where they are well-connected to their peers and family members. By doing so, parents provide their children with key resilience factors, which helps reduce psychiatric vulnerability and ameliorates multiple risk factors for developing psychiatric conditions.

## Data Availability Statement

The raw data supporting the conclusions of this article will be made available by the authors, without undue reservation.

## Ethics Statement

The studies involving human participants were reviewed and approved by Renmin University of China. Written informed consent to participate in this study was provided by the participants' legal guardian/next of kin.

## Author Contributions

DL designed the study. WL and MR collected the data and completed the analyses. JZ and FHZ collected the data. All authors contributed to the article and approved the submitted version.

## Conflict of Interest

The authors declare that the research was conducted in the absence of any commercial or financial relationships that could be construed as a potential conflict of interest.
